# Unraveling the Effects of Epigallocatechin-3-gallate on Hepatocellular Carcinoma Cells: A Comparative Analysis of Monolayer vs Multicellular Tumor Spheroids

**DOI:** 10.1021/acsomega.5c00839

**Published:** 2025-07-31

**Authors:** Mariana dos Reis Simpronio, Ana Rita Thomazela Machado, Patrick Santos, Diego Luis Ribeiro, Lusânia Maria Greggi Antunes, Alexandre Ferro Aissa

**Affiliations:** † Department of Clinical Analysis, Toxicology and Food Sciences, School of Pharmaceutical Sciences of Ribeirão Preto, University of São Paulo—USP, Ribeirão Preto, SP 14040-903, Brazil; ‡ Department of Genetics, Ribeirão Preto Medical School, University of São Paulo—USP, Ribeirão Preto, SP 14049-900, Brazil

## Abstract

The tumor microenvironment is a complex milieu that has not been properly studied in cells cultured in conventional monolayer. Studies have demonstrated the antitumor activity of epigallocatechin-3-gallate (EGCG), present in green tea, using monolayer cultures without considering the three-dimensional microenvironment of a tumor. Furthermore, many studies have shown the effect of EGCG on the transcriptional profile of cancer cells, but each study has been limited to only one or a few cell types. Using the LINCS database, we characterized the gene signatures produced by EGCG treatment in different cell types and reported a variation in EGCG-induced gene signatures depending on the cell type analyzed. GSEA analysis revealed that EGCG influenced multiple biological pathways related to cell signaling, proliferation, epigenetic modifications, and the tumor microenvironment. Then, we cultured hepatocellular carcinoma cells (HepG2) as multicellular tumor spheroids (MTS) to evaluate the effects of EGCG on growth, morphological integrity, cell migration, and cell viability in MTS. We also evaluated the expression of genes related to cell survival and proliferation (*IL6*, *TNF*, *RELA*, *BAX*, *BCL2*), chromatin modification and DNA methylation (*EZH2*, *KDM1A*, *HAT1*, *DNMT3A*), and cell adhesion (*CDH1*, *CD44*, *ITGB2*, *MMP2*). The cell culture condition influenced EGCG effects on gene expression and cell viability, with more significant effects in monolayer than in MTS. After 15 days, control MTS showed cellular dissociation, whereas EGCG-treated MTS showed decreased cell viability and no growth. ECGG blocked the migration of MTS cells into Matrigel and decreased the expression of matrix metalloproteinase *MMP2*. These results suggest that EGCG could prevent cell migration from small nonirrigated tumors *in vitro* by affecting cell adhesion molecules such as MMP2, decreasing the catalytic activity of enzymes associated with metastasis.

## Graphical abstract

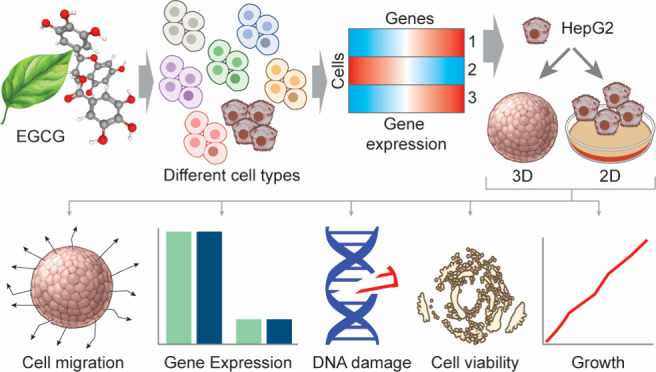

## 1 Introduction

Hepatocellular carcinoma (HCC) is the most common primary liver cancer in adults and the third leading cause of global cancer-related death.^1^ Despite multiple treatments, its incidence is rising due to chemo- and radio-resistance.^2^ Identifying novel targeted therapies is crucial for advanced-stage HCC patients.

Green tea (*Camellia sinensis*) and its polyphenols, especially epigallocatechin-3-gallate (EGCG) (Figure 1), exhibit potent chemopreventive effects.^3,4^ EGCG inhibits HCC cell growth *in vitro* by inducing S phase cell cycle arrest and apoptosis through PI3K/AKT downregulation.^5^ In a thioacetamide-induced HCC model in SD rats, EGCG increased animal survival, reduced liver α-fetoprotein, improved fibrosis, hepatic tissue breakdown, and decreased metalloproteinase-9 (MMP9) expression.^6^ EGCG promotes apoptosis by altering BCL2 and BAX protein levels^7^ and downregulating osteopontin, a protein linked to metastasis and invasion.^8^ Additionally, EGCG induces autophagy in HepG2 cells, leading to reduced secretion and increased degradation of α-fetoprotein, an HCC biomarker.^9^

**Figure 1:**
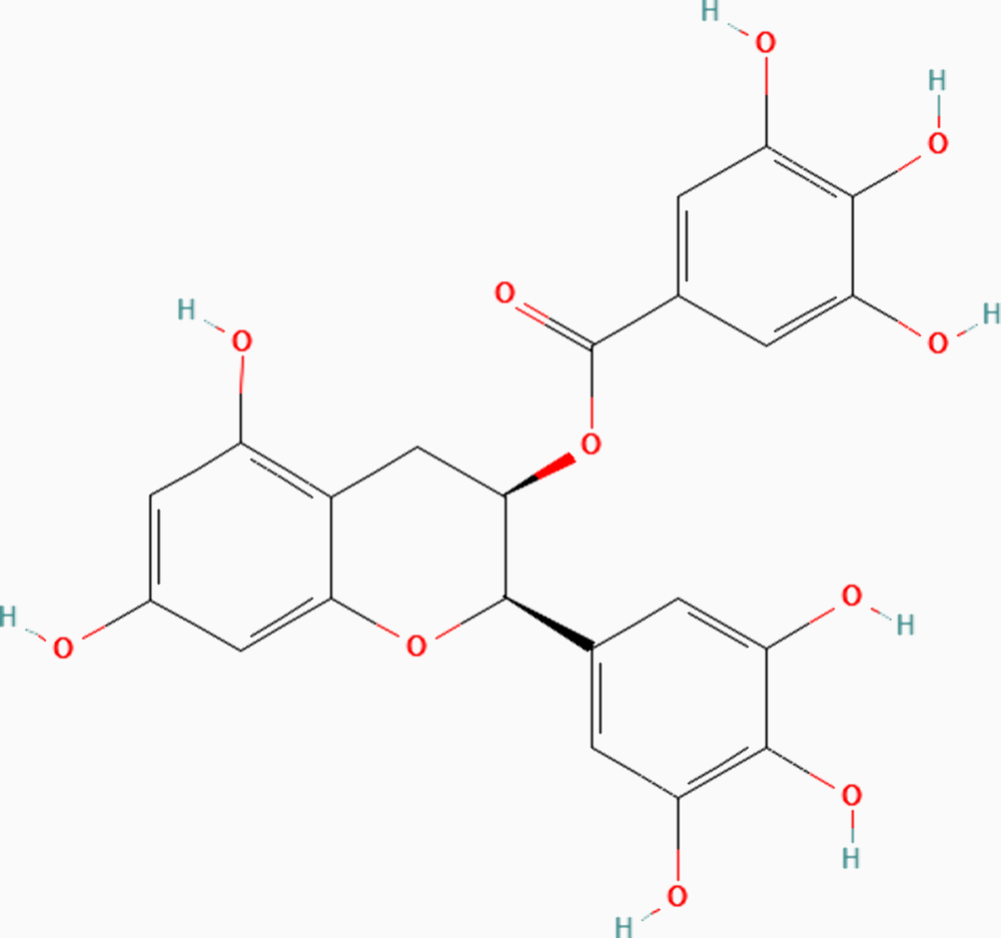
Chemical structure of epigallocatechin-3-gallate (EGCG). Image obtained from PubChem (https://pubchem.ncbi.nlm.nih.gov).

EGCG alters genes related to epigenetic modification and cell proliferation, including chromatin-modifying genes (histone acetyltransferases and histone deacetylases) that control NF-kappa B subunit RELA and its downstream genes, like IL6, involved in cell proliferation and migration.^10–12^ The epigenetic effects of EGCG on cancer chemoprevention have also been investigated through the assessment of global DNA methylation and expression of genes related to DNA methylation, such as DNA methyltransferase enzymes (DNMTs), and genes related to chromatin modification.^13,14^

It has been reported that the activity of EGCG can vary among different tissues and cell lines. Studies have shown that EGCG treatment can either increase or decrease the expression of certain genes depending on the tissue analyzed. For example, Yi et al.^15^ demonstrated that EGCG treatment did not alter IL6 levels in the paraventricular nucleus of the hypothalamus in Wistar rats, but it reduced IL6 levels in SHR rats in the same tissue. Conversely, Wang et al.^16^ found that treating Kunming mice with EGCG increased serum levels of IL6. In KYSE150 cells from human esophageal squamous cell carcinoma, Chen et al.^17^ showed that EGCG treatment did not alter the expression of RELA. However, several other reports have shown that EGCG decreases RELA expression in other cell types such as lymphocytes,^10^ SK-MEL-5 melanoma cells^18^ and normal human bronchial epithelial cells.^19^

Some studies have investigated the impact of EGCG on the transcriptional profile of cancer cells, consistently revealing its ability to modulate gene expression.^20–22^ However, the majority of these studies have been confined to a narrow focus, examining only one or a few specific cell types in isolation. In order to provide a more comprehensive understanding of EGCG’s influence on gene expression across diverse cancer cell lines, a compilation of results from various studies becomes imperative. This approach would not only enhance the breadth of our knowledge but also offer a holistic view of the genes involved, reflecting the intricacies of different cell types. By synthesizing findings from multiple studies, we can better discern commonalities and divergences in EGCG-induced transcriptional changes, paving the way for a more nuanced comprehension of its molecular mechanisms across a spectrum of cancer cell types.

Furthermore, a considerable portion of research studying EGCG was conducted within two-dimensional (2D) cell cultures, however, the investigation did not encompass cell cultures that more accurately depict the complexities of the tumor microenvironment. Three-dimensional (3D) cell culture models, particularly multicellular tumor spheroids (referred to as spheroids), more accurately replicate the tumor microenvironment and crucial *in vivo* processes like gene expression and chemical penetration.^23–25^ Spheroids mimic avascular tumor nodules, micrometastases, or intervascular regions of solid tumors, closely replicating their microenvironment, growth kinetics, and structural architecture.^26,27^ They are essential for predicting drug response and testing antitumor substances due to their ability to simulate chemoresistance observed in solid tumors.^28^ Spheroids also offer a better structure to study cell migration, reflecting changes in cell plasticity necessary for acquiring a migratory phenotype.^29,30^ In monolayer culture, studying cell migration and proliferation separately requires a carefully designed experiment to avoid misinterpretation.^31^

Given the promising antitumor effects of EGCG on HCC cells and its cell-type-dependent gene expression, this study aims to evaluate the antiproliferative, cytotoxic, genotoxic, and antimetastatic effects of EGCG on HepG2 cells in spheroids and monolayer cultures. Additionally, we conduct a comparative gene expression analysis between the two culture conditions, focusing on genes associated with cell proliferation, survival, chromatin modification, and cell adhesion.

## 2 Materials and Methods

### 2.1 Gene Signatures Obtained from the LINCS Database

We used the Library of Integrated Network-based Cellular Signatures (LINCS) portal (https://clue.io/releases/data-dashboard) to obtain gene signatures from level 5 data (level5_beta_trt_cp_n720216x12328.gctx) from different cell lines treated with EGCG (LINCS pert_id: BRD-K55591206).^32,33^ We assigned characteristics related to Cell Source Type, Cell Lineage/Type, Tissue, and Disease for each of the cell types used, using data from LINCS (https://lincsportal.ccs.miami.edu/cells/),^32,33^ DepMap Portal (https://depmap.org/),^34^ Cellosaurus (www.cellosaurus.org),^35^ and American Type Culture Collection (ATCC, www.atcc.org), referenced by the LINCS portal as the supplier of various cell types used (Supplementary Table 1).

We selected the 24 h treatment and 10 μM concentration, as they are the time and concentrations used by LINCS with the highest number of cells and replicates tested. For this purpose, we used the “cmapR” package^36^ to obtain the signatures and the “clusterProfiler” package^37^ to run the Gene Set Enrichment Analysis (GSEA) using Gene Ontology Biological Process (GOBP), Kyoto encyclopedia of genes and genomes (KEGG) pathways, and “Chemical Genetic Perturbations” obtained from Molecular Signatures Database (MSigDB, https://www.gsea-msigdb.org).^38,39^ Only the GSEA with FDR < 0.05 were selected and presented. We then used the package “pheatmap” (https://cran.r-project.org/web/packages/pheatmap/index.html) to build a heatmap (Figure 2) based on the signature values, ranging from low (represented by shades of blue) to high (represented by shades of red). The heatmaps presented in Figure 3 were produced with Gitools version 2.3.1 ^40^ using the matrices generated by the function “GSEA” from the “clusterProfiler” package.

**Figure 2:**
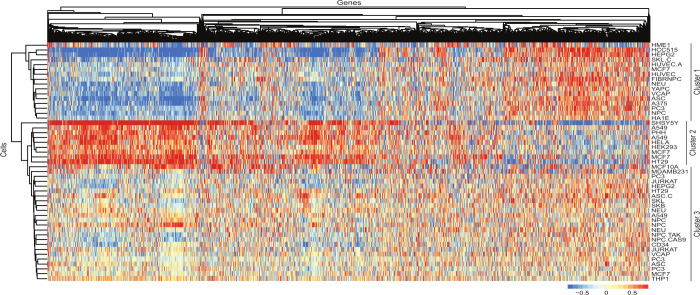
EGCG induces different gene signatures depending on the cell type. Gene signatures obtained from the LINCS database of various cell types (rows) treated with EGCG at 10 μM for 24 h. The clusters on the left were automatically assigned by the “pheatmap” package, and the main three clusters on the right were manually assigned based on the clusters on the left. Blue, low expression; red, high expression.

**Figure 3:**
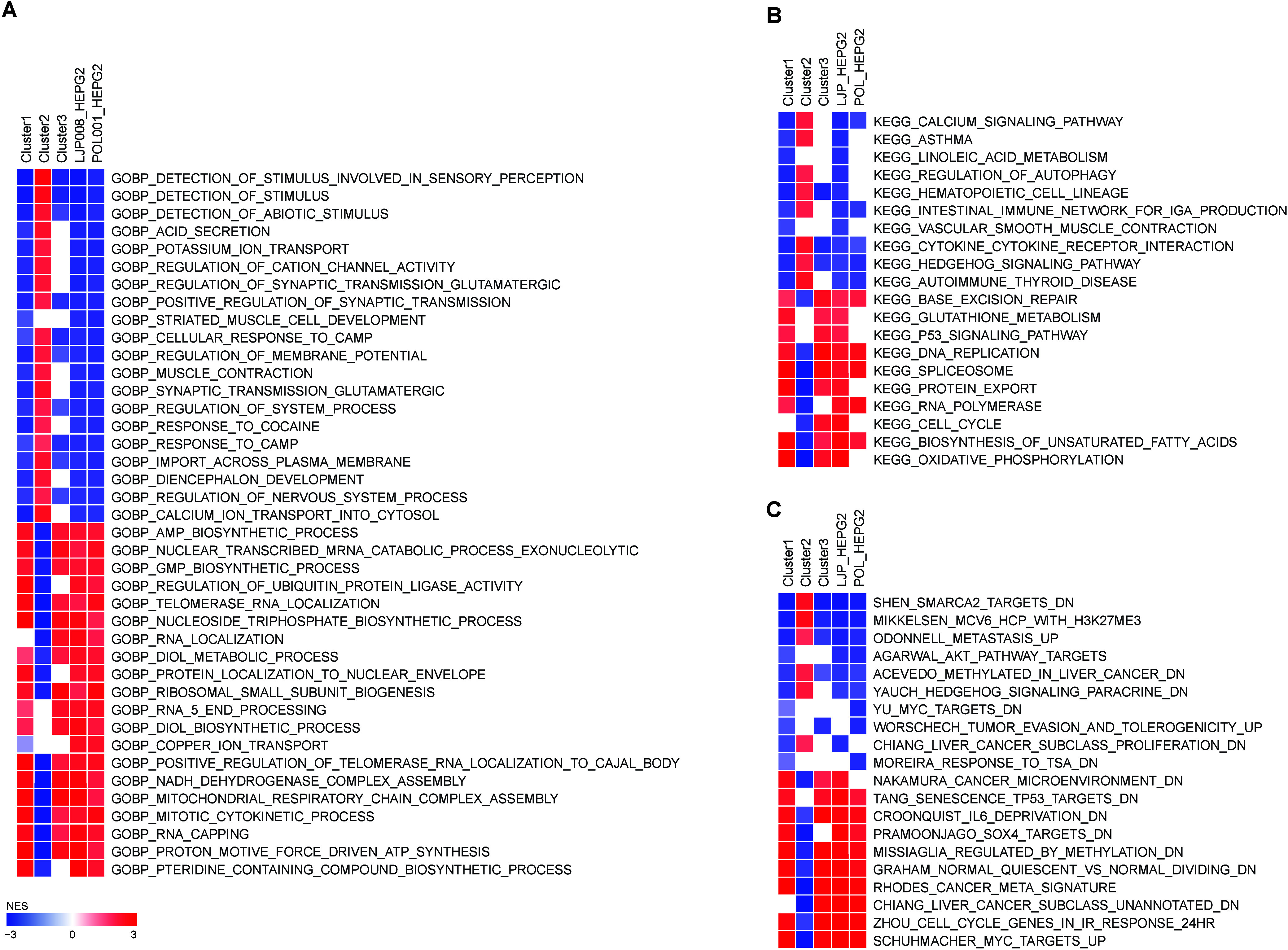
GSEA of the gene signatures produced by EGCG treatment. GSEA in relation to (A) gene ontology biological process (GOBP), (B) KEGG pathways, and (C) Chemical Genetic Perturbations from MSigDB. NES: normalized enrichment score.

### 2.2 EGCG Concentrations

(−)-Epigallocatechin-3-gallate (EGCG; CAS: 989-51-5; C_22_H_18_O_11_) was purchase from Sigma-Aldrich (St. Louis, MO, USA) and promptly dissolved in phosphate buffered saline (PBS,pH 7.4) before the experiments according to its molarity (PM: 458.37 g/L). After, EGCG was kept in sterile tubes, absence of light, and protected from any contact with other reagents to maintain chemical stability. EGCG concentrations were selected based on *in vitro* studies that demonstrated their effect on viability and gene expression of cancer cells including HepG2.^9,41^ Working solutions of 12.5, 25, 50, 100, and 200 μM were diluted in Dulbecco’s Modified Eagle Medium (DMEM, Gibco, Carlsbad, CA, USA) to perform biological tests. All working solutions were prepared 2× more concentrated since the treatments in spheroids consist of replacing 50% of the culture medium. The same aliquots were used to treat spheroids and cells in monolayer.

### 2.3 Cell Line and Culture Conditions

We used human hepatocellular carcinoma HepG2 cell line to perform the experiments in both two and three dimensions. Since the objective of this study is to evaluate the effect of EGCG in two conditions of cells cultures, we decide to focus on the properties of HepG2 that is widely used in studies regarding liver cancer.^42^ HepG2 cell line was obtained from Cell Bank of Rio de Janeiro (BCRJ, Cat. No. 0103), which was authenticated by the vendor. HepG2 cells were maintained in DMEM with 10% Fetal Bovine Serum (FBS; Gibco), 1% antibiotic/antimycotic mix (10,000 units/mL penicillin, 10,000 μg/mL streptomycin and 25 μg/mL amphotericin B; Gibco) and 0.024% sodium bicarbonate (Sigma-Aldrich). Cells used in all experiments were below 10 passages. In cultivation, HepG2 cells were kept in an incubator Thermo Fisher Scientific 3110 Series II CO2 Water Jacketed (ThermoFisher Scientific; Carlsbad, CA, USA) with an atmosphere of 5% CO2 at 37 °C and 96% relative humidity. Spheroids experiments were performed with cell aliquots between the third and eighth passage. All procedures were performed in Class II and typed 1A laminar flow hoods of the Bioprotector VSFL-09 model (VecoFlow Ltd.a; Campinas, SP, Brazil).

### 2.4 Multicellular Tumor Spheroids

Multicellular tumor spheroids were cultured according to Friedrich et al.^26^ Briefly, spheroids initiation was performed with a complete DMEM medium containing HepG2 cells (2 × 10^3^) seeded in 96-well plates (Greiner Bio-One; Monroe, NC, USA) previously coated with 1.5% Normal Melting Point Agarose (NMP; Invitrogen, Carlsbad, CA, USA). Thus, agarose was dissolved in an incomplete DMEM medium and the solution autoclaved for 20 min at 120 °C. After autoclaving, the flask containing the still hot solution was transferred to the class II laminar flow, and the agarose temperature was monitored with a thermometer at 60 °C when it was then pipetted into 96-well plates with Multipette M4 multipipettor (Eppendorf, Hamburg, Germany). After solidification, complete DMEM containing HepG2 cells were added to each well. The same cell suspension was used to assemble a pair of plates containing monolayer cells and spheroids. Thus, each pair of comparisons between the two culture conditions contains the same number of cells and shares the same culturing procedure. The plates were transferred to an incubator with 5% CO_2_ at 37 °C and 96% humidity and held immobile for 4 days (96 h) for the spheroid’s formation. In this procedure, one spheroid is formed in each well.

### 2.5 Area, Morphology, and Integrity Analysis in Spheroids

Area, morphology, and integrity of each spheroid of HepG2 cells were analyzed based on Friedrich et al.^26^ recommendations. After formation (day 0), the first photomicrograph of each spheroid was obtained 72 h after treatment with EGCG. The other photomicrographs were obtained every 48 h until the 15th day. All photomicrographs of spheroids were obtained by the Axio Cam MRc image capture system (Carl Zeiss; Göttingen, Germany) coupled to an inverted Axio LabA1 microscope (Carl Zeiss), using the 10x objective and performing the analysis using the AxioVision 3.1 software (Carl Zeiss). After capturing the photomicrographs, the spheroids were treated with DMEM (negative control) and EGCG (12.5, 25, 50, 100, and 200 μM) by changing 50% of the culture medium. Each spheroids image was scanned to detect irregular spheroids (without circular shape, cellular dissociation, or irregular cell agglomeration). The area measurement of the spheroids was made with the AxioVision 3.1 software using the “Measure” tool. The area is shown in μm^2^.

### 2.6 Cell Viability (Resazurin Reduction Assay)

The resazurin assay (Resazurin sodium salt; Sigma-Aldrich, St. Louis, MO, USA) was performed for cell viability analysis according to Walzl et al.^43^ Briefly, after formation, the HepG2 spheroids were treated with EGCG for 72 h (12.5–200 μM). Cell viability was also analyzed on the 15th day after the last spheroid image capture session. After EGCG treatments, a solution of resazurin 0.5 mM, diluted in PBS, was added to each well for 3 h. Finally, the plate was analyzed on the CaryEclipse Fluorescence Spectrophotometer (Agilent Technologies, Santa Clara, CA, USA) with excitation at λ = 530 nm and emission λ = 590 nm. Fluorescence intensity was used to determine cell viability, comparing the treatment groups with the negative control, which was assigned the value of 100% cell viability.

### 2.7 Comet Assay (Single Cell Gel Electrophoresis)

The evaluation of DNA damage was performed on monolayer and spheroid cultures after 4 h of EGCG treatment, according to the protocol proposed by Olive et al.^44^ with some modifications. For the monolayer culture, cells were washed twice with PBS and incubated with trypsin (Gibco) for 5 min. Then, a complete DMEM medium was added, and the contents of 8 wells were mixed into 1.5 mL tubes. The tubes were centrifuged for 5 min at 300 × *g*, the supernatant discarded, and the resulting pellet resuspended in PBS. For spheroid culture, 80 μL of the culture medium was removed from each well. Then, the remaining contents (120 μL) of eight wells, along with the spheroids, were transferred to a 1.5 mL tube. The tubes were centrifuged for 5 min at 300 × *g*. After that, the supernatant was removed, and trypsin was added to each tube. The tubes were kept in a water bath at 37 °C for 5 min and shaken manually every 1 min. After this period, a complete DMEM culture medium was added and homogenized. Then, each tube was centrifuged for 5 min at 300 × *g*, the supernatant discarded, and the resulting pellet resuspended in PBS. The resulting pellet from each culture condition was resuspended in 0.5% low melting point agarose (LMP; Gibco) 1:4 (v/v; 1 μL of homogenate: 4 μL agarose), and the mixture transferred to a conventional slide precoated with 1.5% NMP and covered with a coverslip (24 × 60 mm). The slides were kept at 4 °C for 10 min. After solidification, the slides were immersed in lysis solution (2.5 M NaCl, 100 mM EDTA, 10 mM Tris, 10% DMSO, 1% Triton X-100, pH 10) for 20–22 h at 4 °C. The slides were treated with alkaline electrophoresis buffer (300 mM NaOH, 1 mM EDTA, pH > 13, 4 °C) for 20 min. Horizontal electrophoresis was performed at 25 V and 300 mA (0.74 V/cm) for 20 min. All steps were conducted without the direct incidence of light. After electrophoresis, the slides were immersed in a neutralization solution (0.4 M Tris, pH 7.5 at 4 °C) for 5 min. The slides were dried at room temperature, fixed in absolute ethanol 99% for 2 min, and, after drying, stored at room temperature until analysis. Before analysis, the slides were stained with 1× GelRed solution (Biotium, Hayward, California, USA). The nucleoids were identified by fluorescence microscopy (AxioStar Plus, Axio Cam MRc, AxioVision 3.1, Carl Zeiss) using 516–560 nm filter, 590 nm filter barrier, and 200× magnification. Images from at least 100 random fields, obtained from two slides (50 nucleoids/slide), were analyzed by the software Comet Assay IV version 4.3 (Perceptive Instruments, Bury St. Edmunds, United Kingdom). The parameter selected for analysis was the intensity of DNA in the tail (Tail Intensity).

### 2.8 Cell Migration in Extracellular Matrix

Analysis of cell migration to the extracellular matrix was done with Matrigel (BD, Franklin Lakes, New Jersey, USA) based on the protocol proposed by Vinci et al.^45^ Initially, Matrigel (10 mg/mL) was diluted in incomplete DMEM to the final concentration of 200 μg/mL, and 50 μL of the solution was pipetted into 96-well plates. Plates were left at room temperature for 3 h to allow Matrigel to solidify and to fix to the wells. After that, the remaining unbound volume was carefully removed, and the wells were washed twice with PBS at room temperature. A blocking solution of 1% bovine serum albumin (BSA) (w/v) diluted in PBS was added. The plates were allowed to stand for 1 h, and after that time, the spheroids were transferred. Next, formed spheroids (after the 96 h initiation period) were transferred to the plates precoated with Matrigel. The EGCG (12.5, 25, 50, 100, and 200 μM) treatments were added. After 30 min incubation, the images of each well corresponding to the time 0 (0h) were acquired. The following images were obtained after 24 and 48 h. For cell migration analysis, each image was evaluated by measuring the circle around the migrated cells using the tool “Measure” in the AxioVision 3.1 software. The area of the spheroid and the cell migration are shown in μm^2^.

### 2.9 Gene Expression (RT-qPCR)

Gene expression experiments were carried out in HepG2 cells cultured in monolayer and spheroid after treatments with EGCG (200 μM) for 72 h following the MIQE guidelines.^46^ A no-template control (NTC) was added, omitting any DNA or RNA template to ensure no extraneous nucleic acid contamination. Cell procedures (trypsinization/collection) for monolayer and spheroids cultures were the same as described in section 2.7. Total RNA extraction was performed using the PureLink RNA Mini Kit (Thermo Fisher Scientific). RNA quantification was conducted in the NanoDrop 200c (Thermo Fisher Scientific). RNA samples with acceptable purity have A_260_/A_230_ ratios between 1.8–2.2 and A_260_/A_280_ equal to 1.8. After this, the cDNA was synthesized with 1 μg of total RNA with the High-Capacity cDNA Reverse Transcription kit (Thermo Fisher Scientific). The primers of the genes *ACTB*, *IL6*, *TNF*, *RELA*, *BAX*, *BCL2*, *EZH2*, *KDM1A*, *HDAC1*, *HAT1*, *DNMT3A*, *CDH1*, *CD44*, *ITGB2*, and *MMP2* were purchased from KiCqStart SYBR Green Primers (Sigma-Aldrich) and are described in Table 1. The reactions were prepared with the Power SYBR Green Master Mix reagent (Thermo Fisher Scientific) and performed in the StepOne Plus Real-Time thermal cycler (Applied Biosystems). Relative expression levels were calculated according to the relative quantification method 2^–ΔΔCt^ proposed by Schmittgen et al.^47^ between the treated groups and the monolayer control group using the gene *ACTB* as the reference gene.

**Table 1:** Sequence of the Primers Used for RT-qPCR

	Primers sequences (5′ → 3′)	
Gene	Foward	Reverse
*ACTB*	GACGACATGGAGAAAATCTG	ATGATCTGGGTCATCTTCTC
*IL*	GCAGAAAAAGGCAAAGAATC	CTACATTTGCCGAAGAGC
*TNF*	CCATGTTGTAGCAAACCC	GAGTAGATGAGGTACAGG
*RELA*	GCAGAAAGAGGACATTGA	GTGCACATCAGCTTGC
*BAX*	AACTGGACAGTAACATGGAG	TTGCTGGCAAAGTAGAAAAG
*BCL2*	GATTGTGGCCTTCTTTGA	GTTCCACAAAGGCATCC
*EZH2*	AAGAAATCTGAGAAGGGACC	CTCTTTACTTCATCAGCTCG
*KDM1A*	CACCGAGTTCACAGTTATTTAG	TAGTTGGTAGGGGTTTTATCC
*HDAC1*	GGATACGGAGATCCCTAATG	CGTGTTCTGGTTAGTCATATTG
*HAT1*	CATGACATGTAGAGGCTTTC	CGTAGCTCCATCCTTATTATAC
*DNMT3A*	TATTGATGAGCGCACAAGAGAGC	GGGTGTTCCAGGGTAACATTGAG
*CDH1*	CCGAGAGCTACACGTTC	TCTTCAAAATTCACTCTGCC
*CD44*	TTATCAGGAGACCAAGACAC	ATCAGCCATTCTGGAATTTG
*ITGB2*	TTGAGAAGGAGAAGCTCAAG	CTAACTCTCAGCAAACTTGG
*MMP2*	GTGATCTTGACCAGAATACC	GCCAATGATCCTGTATGTG

### 2.10 Statistical Analysis

Statistical analysis was done using three independent experiments, each performed with eight technical replicates. For the experiments with only one condition of culture, we used ANOVA, one way, and Dunnet’s test comparing the treated samples against the control. For the experiments with the two conditions of cultures, monolayer, and spheroids, we made multiple comparisons with treated and control samples across the condition of culture using ANOVA, two-way, and Tukey’s multiple comparison test. For the comet assay and gene expression analysis by RT-qPCR, eight spheroids were pooled together to make one out of three replicates (n = 3), that is 24 spheroids in total. For all statistical analyzes, *p* < 0.05 was considered statistically different. All evaluations were performed using the GraphPad Prism 8.0 software (La Jolla, CA, USA).

## 3 Results

### 3.1 EGCG Induces Different Gene Signatures Depending on the Cell Type

In order to verify the gene expression profile of different cell lines treated with EGCG, we used the LINCS database to obtain gene signatures from different cell lines treated with EGCG at 10 μM for 24 h. We then build a heatmap based on the signature values, ranging from low to high. The heatmap revealed the presence of two main clusters where the second could be subdivided into two, creating three large clusters (Figure 1). Importantly, the data set represents a diverse collection of cell types distributed across the three clusters. Cell type classification indicates that clustering was not predominantly driven by Cell Source Type, Cell Lineage/Type, Tissue, or Disease, as cells within these categories are not exclusively confined to single clusters (Supplementary Figure 1 and Supplementary Table 1). This suggests that the clustering captures broader biological diversity rather than being heavily influenced by these specific characteristics. While the distribution is not perfectly uniform, each cluster includes a varied representation of cell types.

The first cluster showed an inverse gene expression pattern in relation to the second cluster; that is, the genes highly expressed in the first cluster were lowly expressed in the second cluster, and vice versa. This result suggests that these two groups of cells have different gene expression profiles and possibly perform other biological functions in response to EGCG treatment. Furthermore, we identified a third cluster that exhibited a gene signature pattern not as clear as the first two and clustered closer to the second cluster but with an expression profile of downregulated genes visually more similar to the first cluster.

### 3.2 Gene Signatures Produced by EGCG Are Related to Cell Signaling and Proliferation, Epigenetics, and Tumor Microenvironment

We then grouped all the cell lines according to the clustering and calculated the average expression of each gene in each cluster. Then we ran a GSEA against the gene ontology biological process (GOBP), KEGG pathways, and “Chemical Genetic Perturbations” (Figure 3). We also added the single signatures profiles of two experiments that used HepG2 cells from hepatocarcinoma.

In general, GSEA analysis showed that the two experiments using HepG2 showed similar enrichment in gene pathways among themselves and similar to clusters 1 and 3, indicating a more remarkable functional similarity between these groups (Figure 3). This result suggests these clusters may perform similar biological functions or be involved in related physiological processes. On the other hand, cluster 2 showed an inverse gene pathway expression pattern in relation to clusters 1 and 3 and HepG2 cells (Figure 3).

Among the GOBP gene sets downregulated by EGCG, there are gene pathways related to the detection of stimulus, regulation of glutamatergic synaptic transmission, regulation of cation channel activity, striated muscle cell development, response to CAMP and regulation of system process, most of them related to cell signaling (Figure 3A). Among the upregulated pathways are the proton motive force driven ATP synthesis, RNA capping, NADH dehydrogenase complex assembly, mitochondrial respiratory chain complex assembly, mitotic cytokinetic process, positive regulation of telomerase RNA localization to cajal body pathways, most of them related to energy production and cell division (Figure 3A).

Regarding the KEGG pathways, we observed that the downregulated genes were related to the calcium signaling pathway, linoleic acid metabolism, regulation of autophagy, vascular smooth muscle contraction, cytokine receptor interaction, and the hedgehog signaling pathway (Figure 3B). Among the upregulated pathways there are those related to oxidative phosphorylation, biosynthesis of unsaturated fatty acids, cell cycle, RNA polymerase, protein export, and base excision repair (Figure 3B).

We also evaluated these signatures against “Chemical Genetic Perturbations” signature sets, which represent sets of genes up or downregulated by perturbants (Figure 3C). In this case, downregulated pathways were associated with SMARCA2 targets, epigenetic changes, metastasis, AKT targets, Hedgehog signaling, MYC targets, tumor evasion, and liver cancer. Upregulated pathways were associated with MYC targets, cell cycle, liver cancer, dividing cells, methylation, SOX4 targets, IL6 deprivation, senescence and tumor microenvironment (Figure 3C).

These data suggest that HepG2 cells resemble a broader group of cell lines that exhibit a coordinated response to EGCG treatment, characterized by the downregulation of pathways related to cell signaling and invasion, and the upregulation of pathways associated with energy metabolism, cell cycle, epigenetic changes, metastasis, transcription factors linked to proliferation, tumor evasion, and liver cancer. Based on these findings, we experimentally investigated the effects of EGCG on some of these processes in HepG2 cells cultured in monolayer or as multicellular tumor spheroids.

### 3.3 EGCG Maintained the Tumor Cells Entrapped in the Spheroids while Decreasing Cell Viability

Initial in silico analyses showed that the effect of EGCG is dependent on the cell type and generates gene signatures that may be related to cell plasticity and the tumor microenvironment. Cells cultured in three dimension show different responses to stimuli when compared to cells cultured in a conventional monolayer.^48^ In this scenario, multicellular spheroids have been used since they could mimic the tumor microenvironment.^49^ Therefore, we selected HepG2 cells from hepatocellular carcinoma to evaluate the effects of EGCG on cells cultured in two and three dimensions in the form of multicellular tumor spheroids.

First, we compared the cell viability of different concentrations of EGCG in HepG2 cells cultured in monolayer or as spheroids (Figure 4A) for 3 days. Cells grown in monolayer presented lower cell viability when compared to cells cultured in spheroids. In fact, the effect of EGCG on the cell viability of spheroid-cultured cells was not significant. The monolayer cells treated with 12.5, 25, 50, 100, and 200 μM of EGCG showed 82, 87, 76, 66, and 54% of cell viability of the control. In contrast, cells cultured as spheroids showed 85, 88, 92, 90, and 89% of cell viability of the control, respectively (Figure 4A). These results indicate that cells cultured in spheroids have less sensitivity to EGCG. However, EGCG treatment for 15 days profoundly affected cell viability on HepG2 spheroids (Figure 4 B). EGCG concentrations of 50, 100, and 200 μM significantly decreased cell viability to 81, 56, and 23%, respectively (Figure 4 B). These findings highlight the potential antitumor activity of EGCG, particularly in reducing the viability of cells within a 3D tumor-like microenvironment.

**Figure 4:**
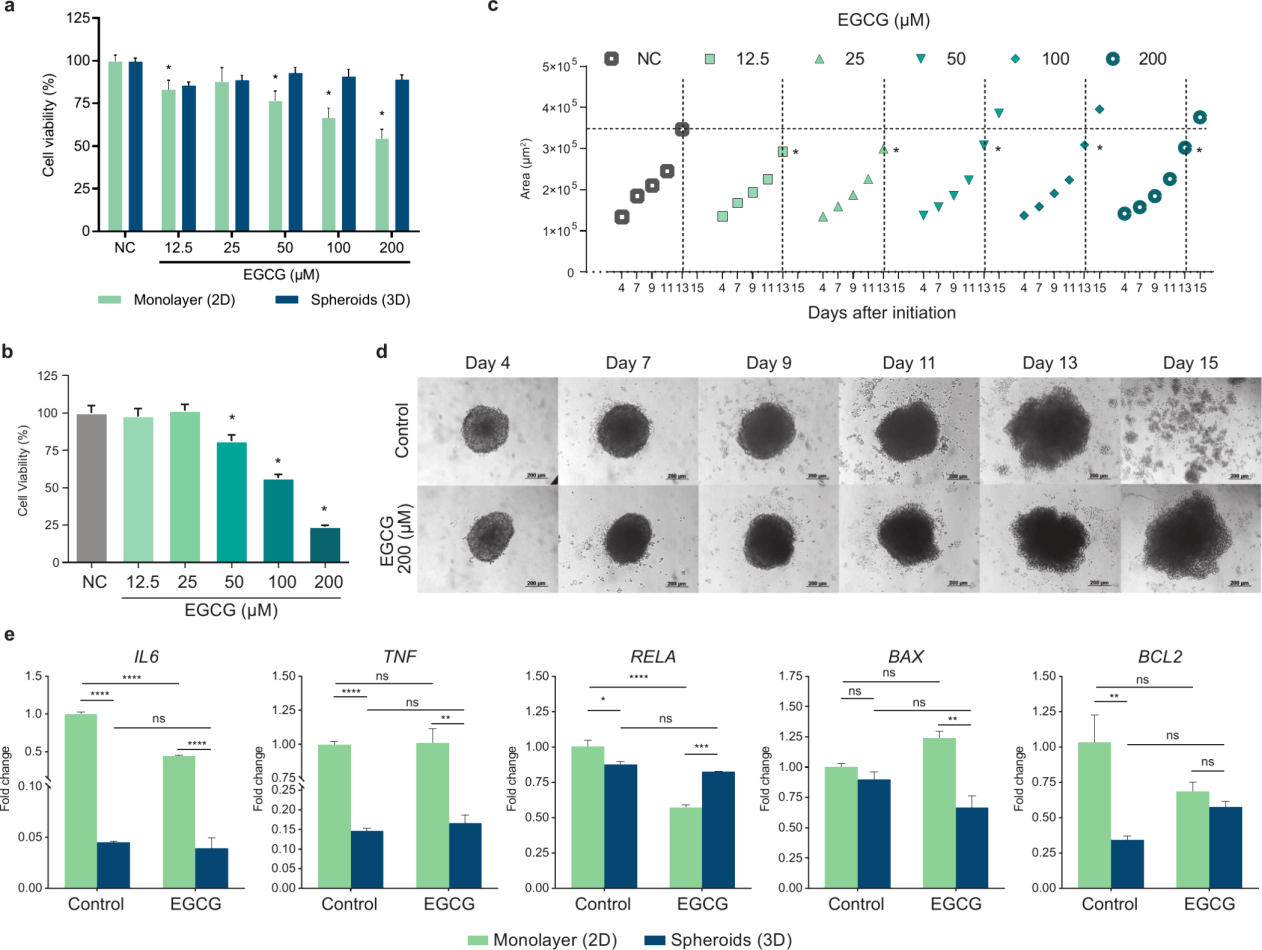
EGCG maintained the tumor cells entrapped in the spheroids while decreasing cell viability. (a) Viability of HepG2 cells cultured as monolayer or spheroids treated with epigallocatechin-3-gallate (EGCG) for 72 h, assessed by the resazurin reduction assay. Data represent mean ± SD (*n* = 8). *Statistically different from the control (PBS) of the respective culture condition. ANOVA, two-way, and Dunnet’s multiple comparison test (*p* < 0.05). (b) Viability of HepG2 cells cultured as spheroids treated with epigallocatechin-3-gallate (EGCG) for 15 days, assessed by the resazurin reduction assay. Data represent mean ± SD (*n* = 8). *Statistically different from the control (PBS). ANOVA, one-way, and Dunnet’s multiple comparison test (*p* < 0.05). (c) Growth of the spheroids evaluated for 15 days after treatment with epigallocatechin-3-gallate (EGCG). The dots represent the mean area covered by the spheroids (*n* = 8). (d) Representative images of the growth of the spheroids as in (b). (e) Relative expression of genes associated with cell proliferation and survival in HepG2-2 cells cultured as monolayer or spheroids treated with 200 μM epigallocatechin-3-gallate (EGCG) for 72 h. *ACTB* was used as the reference gene for normalization relative to control monolayer cells. Data represent mean ± SD (*n* = 3). ANOVA, two-way, and Tukey’s multiple comparison test (*p < 0.05; **p < 0.01; ***p < 0.001; ****p < 0.0001).

We measured the growth, morphology, and integrity of the spheroids treated with different concentrations of EGCG for 15 days (Figure 4C,D). Until day 11, no difference in spheroid size was observed between the different concentrations of EGCG and the control. On day 13, spheroids treated with EGCG presented a smaller size than the control (*p* < 0.05). On day 15, spheroids treated with the control and the lowest concentrations of EGCG (12.5 and 25 μM) showed cellular dissociation. In contrast, spheroids treated with 50, 100, and 200 μM of EGCG showed sustained growth and normal integrity (Figure 4C,D). Interestingly, cells treated for 15 days with EGCG at 12.5 and 25 μM showed ∼100% cell viability, while EGCG at concentrations of 50, 100, and 200 μM decreased cell viability to 81, 56, and 23%, respectively (Figure 4B). This suggests that the higher concentrations of EGCG decreased the viability of HepG2 spheroids, in addition to the prevention of the dissociation of tumor spheroids.

Spheroids are characterized by cells with low proliferative capacity within the core of the sphere, where cells experience a hypoxic environment and a quiescent state.^50^ For this reason, we measured the expression of the cell proliferation-related genes *IL6*, *TNF*, *RELA*, *BAX*, and *BCL2* (Figure 4E). In monolayer cells, EGCG treatment significantly decreased the expression of *IL6* and *RELA*, indicating an impact on genes associated with proliferation in this culture model. However, in spheroids, EGCG treatment did not significantly alter the expression of these genes, possibly reflecting the lower proliferative capacity of cells in this culture model. Additionally, the expression of *IL6* and *TNF* was noticeably lower in spheroids, regardless of treatment, compared to the monolayer culture.

In relation to the pro-apoptotic gene *BAX*, its expression was not changed by the treatment but was lower in spheroids compared to the monolayer. The antiapoptotic gene *BCL2* showed reduced expression in control spheroids compared to the control monolayer and was not influenced by EGCG treatment. These findings suggest that the spheroid model reflects a more quiescent phenotype, where the effects of EGCG on proliferation-related gene expression are less pronounced than in the monolayer culture. This supports the relevance of using spheroids to measure the impact of substances on gene expression in a microenvironment that more closely resembles in vivo conditions.

### 3.4 EGCG Effects in the Expression of Genes Associated with Chromatin Modification and DNA Methylation Is Dependent on the Condition of Cell Culture

To assess the influence of EGCG on the expression of genes related to epigenetics, we evaluated the expression of the DNA methyltransferase gene *DNMT3A,* genes responsible for histone acetylation (*HAT1*) and deacetylation (*HDAC1*), and histone methylation (*EZH2*) and demethylation (*KDM1A*). We found that EGCG downregulated *EZH2 and DNMT3A* only in the monolayer culture but not in the spheroids (Figure 5A). On the other hand, *HDAC1* was downregulated by EGCG only in the spheroids but not in the monolayer culture. Interestingly, the expression of *KDM1A*, *HDAC1*, and *HAT1* was higher in spheroids than in the monolayer model in both control and EGCG-treated cells. *EZH2* and *DNMT3A* expression were also higher in spheroids than in monolayer but only in EGCG-treated cells.

**Figure 5:**
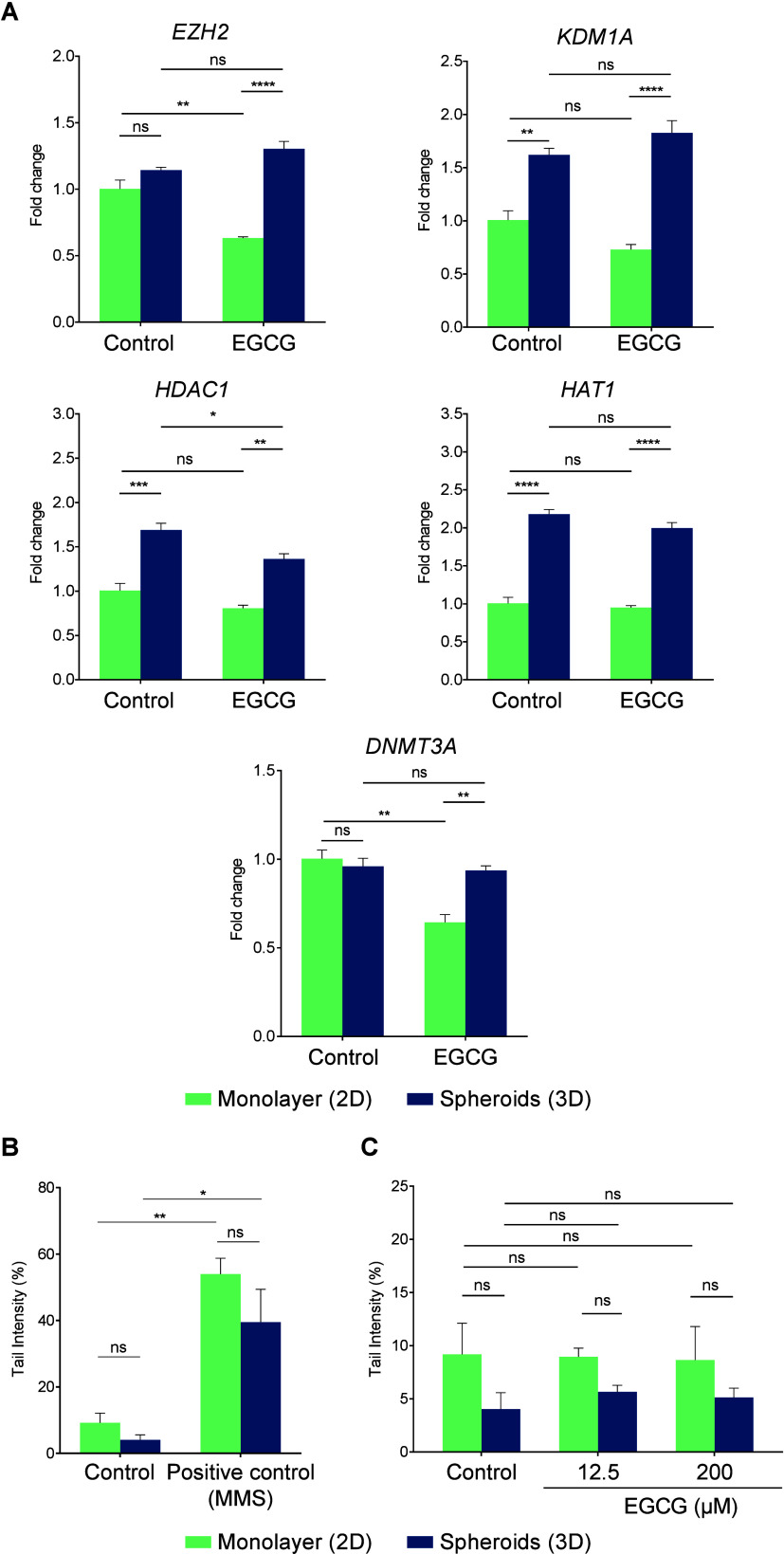
EGCG effects in the expression of genes associated with chromatin modification and DNA methylation are dependent on the condition of cell culture. (A) Relative expression of genes associated with chromatin modification and DNA methylation in HepG2-2 cells cultured as monolayer or spheroids treated with 200 μM epigallocatechin-3-gallate (EGCG) for 72 h. *ACTB* was used as the reference gene for normalization relative to control monolayer cells. Data represent mean ± SD (*n* = 3). ANOVA, two-way, and Tukey’s multiple comparison test (*p* < 0.05; **p < 0.01; ***p < 0.001; ****p < 0.0001). (B) Tail intensity of nucleoids of HepG2 cells cultured as monolayer or spheroids treated with methylmethanesulfonate (MMS), used as a positive control, for 4 h. Data represent mean ± SD (*n* = 3). ANOVA, two-way, and Tukey’s multiple comparison test (*p < 0.05; **p < 0.01). (C) Tail intensity of nucleoids of HepG2 cells cultured as monolayer or spheroids treated with epigallocatechin-3-gallate (EGCG) for 4 h. Data represent mean ± SD (*n* = 3). ANOVA, two-way, and Tukey’s multiple comparison test (ns = not significant).

Since chromatin-modifying genes participates in DNA damage response by regulating chromatin remodeling to prepare the damaged site for repair,^51^ we used comet assay to assess whether the condition of culture or treatment with EGCG induces DNA damage (Figure 5C). Cells treated with the positive control methylmethanesulfonate presented DNA damage, with 54% damage in cells grown in monolayers and 39% in cells cultured in spheroids (Figure 5B).

EGCG neither induced nor reduced DNA damage compared to the control group in either culture condition (Figure 5C). The culture system also did not influence the baseline levels of DNA damage. It is important to note that, although we did not detect DNA damage under the tested conditions, we acknowledge that the version of the comet assay used has limitations and may not detect all types of lesions, particularly oxidative damage.^52^ Therefore, we suggest that future studies employ modified versions of the comet assay capable of identifying more subtle DNA lesions. Nonetheless, based on our results, we conclude that the EGCG-induced modulation of chromatin-modifying gene expression in our model is likely not associated with DNA damage.

### 3.5 EGCG Decreased the Migration of Cells from Spheroid to Extracellular Matrix

Spheroids offer a satisfactory model for cell migration because of their three-dimensional structure of the aggregated cells and their interaction with the extracellular matrix proteins.^45^ Accordingly, we measured the spheroid growth and cell migration after EGCG treatments for 24 and 48 h (Figure 6). There was no significant change in spheroid growth 48 h after treatment with EGCG (Figure 6A and B). At the end of the last analysis (48 h), cell viability analysis showed that only the concentration of 200 μM EGCG reduced cell viability (Figure 5C). Regarding cell migration, after 24 h of treatment, spheroids treated with 100 and 200 μM of EGCG showed a reduction in cell migration (Figure 6D). After 48 h, EGCG at concentrations of 50, 100, and 200 μM reduced migration, with a more expressive effect at 200 μM, which completely inhibited cell migration to the extracellular matrix (Figure 6D).

**Figure 6:**
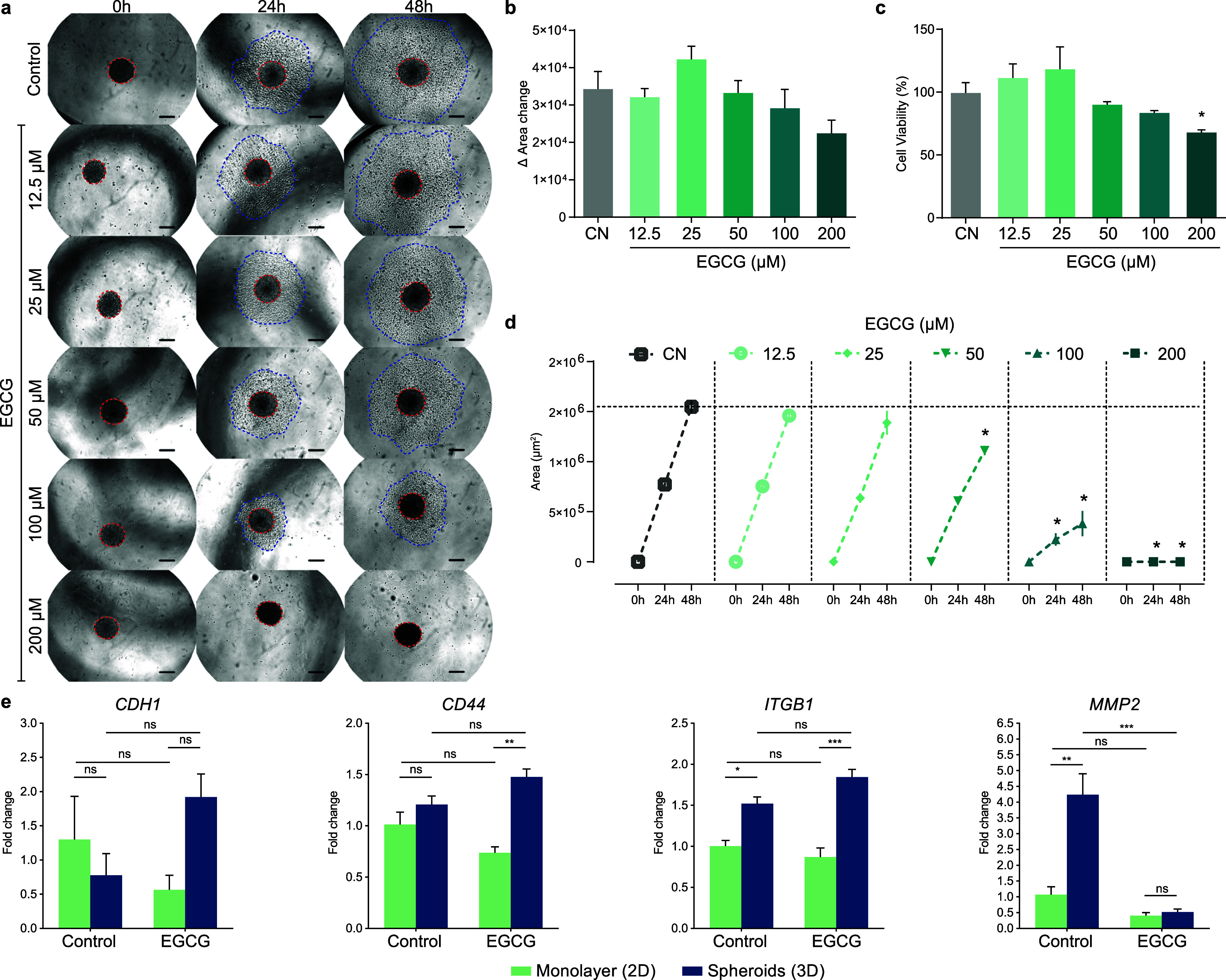
EGCG decreased the migration of cells from spheroid to extracellular matrix. (a) Representative images of the spheroid growth (red dashed lined) and cell migration (blue dashed lined) on the extracellular matrix (Matrigel), evaluated at 0, 24, and 48 h after treatment with epigallocatechin-3-gallate (EGCG). Original increase of 10×. Scale: 200 μm. (b) Change of the area covered by the spheroid as shown in (a) (red dashed lined) 48 h after treatment with epigallocatechin-3-gallate (EGCG). Data represent mean ± SD (*n* = 8). *Statistically different from the control (PBS). ANOVA, one-way, and Dunnet post-test (*p* < 0.05). (c) Cell viability after 48 h, assessed by the resazurin reduction assay. Data represent mean ± SD (*n* = 8). *Statistically different from the control (PBS). ANOVA, one-way, and Dunnet post-test (*p* < 0.05). (d) Area of cell migration to the extracellular matrix (Matrigel), evaluated at 0, 24, and 48 h after treatment with EGCG. The dots represent the area covered by the migrated cells as shown in (a) (blue dashed lined) (*n* = 8). *Statistically different from the control (PBS). ANOVA, one-way, and Dunnet post-test (*p* < 0.05). (e) Relative expression of genes associated with cell adhesion in HepG2 cells cultured as monolayer or spheroids treated with 200 μM epigallocatechin-3-gallate (EGCG) for 72 h. *ACTB* was used as the reference gene for normalization relative to control monolayer cells. Data represent mean ± SD (*n* = 3). ANOVA, two-way, and Tukey’s multiple comparison test (*p < 0.05; **p < 0.01; ***p < 0.001).

Since cell plasticity is often controlled by gene expression changes triggered by the cells in response to external stimulus,^53^ we measured the expression of critical genes associated with cell adhesion and migration (Figure 6E). *CDH1* and *CD44*, which are related to cell adhesion and epithelial–mesenchymal transition,^54,55^ were not influenced by EGCG treatment (Figure 6E). However, *CD44* was increased in spheroids compared with monolayer, both treated with EGCG. We also measured the expression of *ITGB1*, which can bind to extracellular matrix proteins and plays a crucial role in adhesion between cells and the extracellular matrix.^56^ EGCG did not alter the expression of *ITGB1* neither in spheroids nor in monolayer cells. Interestingly, despite treatment, *ITGB1* expression was higher in spheroids than monolayer cells. The most expressive alteration was observed in the metalloproteinase gene *MMP2*, responsible for the proteolysis of extracellular matrix structural proteins under physiological and pathological conditions.^57^ *MMP2* was upregulated by 4.23-fold in control spheroids compared with control monolayer cells. However, when we compare the control spheroids with the EGCG-treated spheroids, there is a marked difference in the expression of *MMP2*, suggesting that EGCG suppressed the gene expression of the gene when cultured in spheroids.

## 4 Discussion

The activity of compounds on molecular mechanisms depends on cell type.^58,59^ The LINCS L1000 project characterizes gene expression profiles for diverse cell types treated with perturbants, creating gene signatures for studied molecules.^32,33^ Gene expression variation post-treatment is influenced by the cell type and microenvironment, notably in tumors, where the tumor microenvironment includes cells of the same type, possibly with different acquired mutations, and other supportive cell types.^60,61^

Using the LINCS database, we reported EGCG-induced gene signatures. LINCS data showed that EGCG’s gene expression profile varies with the evaluated cell condition. Some cells exhibit an inverse gene expression pattern, with a gene downregulated in one cell condition and upregulated in another. These findings highlight the importance of considering cell condition in assessing EGCG effects.

Via GSEA analysis, inferring functions related to differential gene expression groups, EGCG influences various pathways, particularly in cell signaling, proliferation, epigenetic modifications, cell plasticity, tumor microenvironment, and metastasis. Specifically, we identified pathway enrichment in stimulus detection, receptor activation for chemical or physical signals, regulation of glutamatergic synaptic transmission crucial for neuronal communication, control of cation channel activity across the cell membrane, development of striated muscle cells, response to cyclic AMP (cAMP) as a significant second messenger in signal transduction, and regulation of systemic processes encompassing various aspects of organismal functioning.

Analyzing chemical and genetic perturbations proved insightful for characterizing EGCG’s effects on cellular mechanisms previously validated in studies with other compounds. Pathways related to hedgehog signaling, tumor evasion, and the tumor microenvironment may be connected to the activation of genes controlling cell plasticity, typically associated with drug resistance, migration, and invasion.^53^ These results guided *in vitro* experiments to further characterize EGCG effects on HepG2 hepatocellular carcinoma cells, cultured as monolayers or spheroids.

It has been shown that EGCG can induce cell death in various types of cancer cells *in* vitro.^4^ This cytotoxic effect was confirmed in HepG2 monolayer cultures, but not in spheroid cultures, indicating greater EGCG tolerance in the 3D model. One possible explanation is acquired drug tolerance associated with hypoxia, a condition also observed with chemotherapeutics such as cisplatin and doxorubicin.^62^ Tumor spheroids mimic several features of in vivo tumors, including the presence of oxygen and nutrient gradients that generate distinct proliferative, hypoxic, and necrotic zones.^63^ These gradients evolve with spheroid size: spheroids around 200 μm typically contain proliferative normoxic cells, while those exceeding 500 μm display stratification into a proliferative outer layer, a quiescent intermediate zone, and a hypoxic/necrotic core.^64^

Such spatial heterogeneity can influence both gene expression and drug sensitivity, potentially contributing to the observed differential response to EGCG in spheroids versus monolayers. Although our study did not directly measure oxygen or nutrient levels, the reduced effect of EGCG in spheroids may reflect limited drug penetration, altered metabolic states, or the activation of hypoxia-induced survival pathways. Therefore, while 3D cultures better replicate the structural and physiological features of tumors, they also introduce complexity and variability that must be carefully considered when interpreting gene expression and treatment outcomes. Nonetheless, these very features make spheroids a valuable tool for studying therapeutic resistance and tumor behavior under more physiologically relevant conditions.

The observed variations in response between monolayer cells and spheroids may also reflect distinct nutrient uptake dynamics intrinsic to each culture system. Spheroids often exhibit diffusion limitations that can alter metabolic activity, affecting both drug availability and cellular responses to treatment. Although our study did not directly assess nutrient concentrations or metabolic fluxes, previous reports have documented reduced nutrient accessibility in 3D cultures and its impact on therapeutic efficacy.^65–68^ These considerations underscore the relevance of including metabolic parameters in future studies to better understand the interplay between microenvironmental constraints and EGCG sensitivity.

After 15 days, spheroids not treated with EGCG or treated with the lowest concentrations exhibited cellular dissociation, while those treated with 50, 100, and 200 μM EGCG demonstrated preserved structure, reduced growth, and decreased cell viability with increasing EGCG concentration. This suggests that EGCG may act on cell–cell or cell-matrix adhesion, preventing dissociation despite reduced cell viability, a phenomenon consistent with previous studies on EGCG’s influence on adhesion molecules. Spheroid dissociation mimics tissue dissociation, a model anticipating in vivo cell invasion.^69^ Metastatic cascade steps include angiogenesis, cadherin- and catenin-mediated tumor cell dissociation, and invasion through the tumor epithelium’s extracellular matrix.^70^ In this study, spheroid dissociation in the control group may reflect natural processes in 3D cultures, such as nutrient and oxygen depletion, leading to reduced cohesion and structural breakdown over time. EGCG appears to counteract these effects and act on initial metastasis steps, hindering tumor cellular dissociation. While EGCG’s reduction of cancer cell adhesion is established in monolayer cultures,^71^ its effects in the three-dimensional cell culture model remain unexplored and warrant further investigation into the expression of cell integrity-related proteins such as cadherins or integrins.

*IL6* and *TNF* were reduced in spheroids compared to monolayer cells. *TNF*, produced by tumor cells, promotes the growth and spread of tumors in various tissues.^72–77^ IL6, known for its pro-tumor activity, has been implicated in liver and various cancer types.^78,79^ IL6, functioning as a regulator of the acute inflammatory response, activates STAT3, leading to the transcriptional activation of cell survival-related genes.^80^ Proliferative cancer cells typically exhibit increased *IL6* and *TNF* expression, as seen in monolayer cells, but not in spheroids. EGCG decreased *IL6* expression in monolayer cells but not in spheroids. While EGCG’s downregulation of IL6 and TNF in inflammation models and cancer cells is documented,^81^ its impact in spheroids is a novel exploration. These findings underscore the importance of using cellular models that better mimic the *in vivo* environment when studying compound influences on cancer cell gene expression.

Compounds can modulate chromatin conformation, impacting cellular processes.^10,82^ Dysregulation of chromatin-modifying enzymes can affect DNA damage response by altering chromatin assembly at damage sites, gene expression of DNA damage response genes, or participating in DNA repair via nonhomologous end-joining.^83^ Multicellular tumor spheroids mimic the tumor microenvironment, inducing oxygen tension favoring DNA damage. EGCG did not induce DNA damage, as evidenced by the comet assay, suggesting chromatin-modifying gene changes may stem from EGCG’s direct impact on epigenetic mechanisms. EGCG downregulated *EZH2, KDM1A,* and *DNMT3A* only in monolayer cells, consistent with previous reports on EZH2 and DNMTs. These genes remained unaltered in spheroids, indicating greater EGCG tolerance in spheroid culture, similar to cytotoxicity findings. *HDAC1* was downregulated exclusively in spheroids, differing from prior studies. Notably, baseline *HDAC1* levels were higher in spheroids, potentially explaining EGCG’s greater efficacy in downregulating *HDAC1* in spheroids compared to monolayer cells.

Another limitation of our study is the lack of direct analysis of chromatin alterations at the protein level. While we demonstrate EGCG-mediated modulation of mRNA expression for key epigenetic enzymes (e.g., EZH2, DNMT3A, KDM1A, HDAC1), we did not assess whether these transcriptional changes translate into functional modifications in histone marks. Specifically, evaluating changes in histone methylation (e.g., H3K27me3) and total histone levels using techniques such as Western blot or ChIP from nuclear extracts would provide valuable mechanistic insight. Our current approach focused solely on gene expression related to chromatin regulation, which suggests potential structural alterations but does not confirm them. We acknowledge this as an important limitation and emphasize the need for future studies to explore EGCG-induced epigenetic remodeling in both monolayer and spheroid culture models.

Concerning DNA damage, consistent with other findings here, spheroids exhibited less baseline damage than monolayer cells at the lowest EGCG concentration (12.5 μM). This suggests that DNA damage is not the mechanism through which EGCG diminishes tumor cell viability. While three-dimensional model studies on EGCG effects are lacking, existing research indicates that, under specific conditions, EGCG can protect cells from DNA damage induced by genotoxic agents. Johnson and Loo^84^ demonstrated EGCG’s protective effect against oxidative DNA damage induced by H2O2 in Jurkat cells, with 10 μM sufficient for protection, and 100 μM exacerbating damage. In our study, all EGCG concentrations (12.5–200 μM) failed to induce DNA damage. Kanwal et al.^85^ proposed a mechanism involving EGCG’s potential protection against oxidative DNA damage. Using LNCaP cells, they showed EGCG treatment led to the re-expression of the GSTP1 gene, previously inactivated by siRNA, potentially influencing protection against oxidative DNA damage caused by H2O2. Additionally, EGCG has been demonstrated to dose-dependently increase the expression of NRF2/NFE2L2-regulated antioxidant genes, including GST and NQO1, in endothelial cells.^86^.

EGCG influenced spheroid cell migration, evident in the Matrigel migration assay, with complete inhibition at the highest concentration. Metastasis relies on cell movement, making drugs that impact cancer cell motility an attractive therapeutic strategy.^87^ EGCG has been shown to dose-dependently inhibit cell motility, increase cell stiffness, induce rigid cell membrane elasticity, and inhibit vimentin and Slug (SNAI2) transcription factor expression in human lung cancer cells.^88^ Punathil et al.^89^ investigated molecular mechanisms involved in EGCG-induced reduction of cancer cell migration, highlighting the blockade of l-arginine’s migration-promoting capacity, reduction of elevated cGMP levels, re-establishment of 8-Br cGMP (cGMP analog) activity, and inhibition of 4T1 migration through nitric oxide/nitric oxide synthases and guanylate cyclase inhibition.

EGCG maintains HepG2 spheroid cellular aggregation but does not impede growth despite reducing viability and inhibiting cell migration to the extracellular matrix. These results highlight EGCG’s impact on intercellular connections in three-dimensional cell cultures. Examining gene expression associated with cell adhesion, we observed divergent *CD44* gene responses between monolayer and spheroid cultures treated with EGCG. CD44 mediates interactions between tumor cells and the extracellular matrix through binding with hyaluronic acid.^90^ Annabi et al.^91^ demonstrated EGCG inhibiting hyaluronic acid binding to CD44 in U-87 glioma cells, particularly when treated with type-I collagen. Furthermore, treating glioma cells with EGCG reduces CD44 shedding.^92^ In spheroids, where hyaluronan synthases activity is high, inhibiting these enzymes prevents spheroid formation.^93^ Therefore, EGCG-induced *CD44* increase in spheroids may serve as a compensatory mechanism for inhibited hyaluronic acid binding.

EGCG maintained *ITGB1* gene expression, but upregulated it in spheroids compared to monolayer cells. *ITGB1*, a cell-surface receptor pivotal for proliferation, migration, invasion, and survival,^94^ exhibited altered expression in spheroids, aligning with studies highlighting distinct gene expression profiles in three-dimensional versus two-dimensional cell cultures, particularly in genes associated with cell adhesion.^24^

There was a marked decrease in *MMP2* gene expression, an enzyme cleaving extracellular matrix components contributing to invasiveness and metastasis.^95^ Notably, in conventional monolayer culture, cells showed no *MMP2* expression alteration. Increased MMP2 expression is associated with poor overall survival in colorectal cancer patients.^96^ These findings suggest EGCG might hinder cell migration from small nonirrigated tumors by influencing cell adhesion molecules like MMP2, thereby reducing catalytic activity associated with metastasis.

A recent study using reverse molecular docking identified potential EGCG targets across the human proteome, including proteins such as KRAS, CDK2, MMP1, and PIM1, many of which are involved in cell proliferation, extracellular matrix remodeling, and epigenetic regulation.^97^ These findings are consistent with the gene expression changes observed in our study, particularly the downregulation of *MMP2*, *EZH2*, *DNMT3A*, and *HDAC1*. The identification of MMP1 and PIM1 as potential targets reinforces the hypothesis that EGCG may modulate cell adhesion and invasion through direct or indirect effects on these pathways, supporting its multifaceted action on the tumor microenvironment.

Taken together, our findings suggest a mechanistic link between EGCG-induced epigenetic modulation and suppression of pro-metastatic behaviors in tumor spheroids. The downregulation of *MMP2* specifically in spheroids, but not in monolayer cultures, indicates that EGCG may inhibit metastatic potential in tumor-like environments by targeting extracellular matrix remodeling.^98^ This effect may be mediated through chromatin remodeling, as EGCG also modulated the expression of genes such as *HDAC1* in spheroids and *EZH2*, *DNMT3A*, and *KDM1A* in monolayers.^98,99^ These epigenetic regulators influence gene accessibility and may indirectly regulate transcriptional programs involved in migration and invasion. Thus, our data support a model in which EGCG exerts antimetastatic effects through coordinated regulation of chromatin state and adhesion-related gene expression within the 3D tumor microenvironment.

It is important to acknowledge that the EGCG concentrations used in this study are higher than those typically reached through regular dietary consumption of green tea. However, such concentrations are within the range of those achievable through pharmacological supplementation, as previously reported in studies using encapsulated or nanoparticle-based delivery systems.^100,101^ Therefore, while our findings may not directly translate to the effects of tea consumption alone, they are relevant in the context of therapeutic EGCG formulations. These results support the rationale for further studies investigating EGCG as an adjuvant in cancer treatment, potentially in combination with conventional chemotherapeutics, and highlight the need for clinical trials exploring its pharmacokinetics, bioavailability, and safety in therapeutic doses.

## 5 Conclusion

Using the LINCS database, we examined the gene signatures resulting from EGCG treatment in various cell types. We observed distinct variations in EGCG-induced gene signatures based on the analyzed cell type. GSEA analysis further identified the impact of EGCG on multiple biological pathways associated with cell signaling, proliferation, epigenetic modifications, and the tumor microenvironment. Cells grown as spheroids showed less sensitivity to EGCG than HepG2 cells cultured in a monolayer. EGCG treatment also decreased cell viability and prevented the spheroid from rupturing, which could release cells with the invasive phenotype to adjacent tissues. EGCG altered the expression of genes associated with cell proliferation. However, this effect was more pronounced in monolayer cells than in spheroids, which supports the relevance of using spheroids to measure the impact of therapeutic agents on the gene expression of cancer cells. EGCG blocked cell migration from spheroids, accompanied by a reduction in the expression of matrix metalloproteinase gene *MMP2*, responsible for the degradation of the extracellular matrix associated with metastasis. Interestingly, EGCG did not alter the expression of *MMP2* in monolayer cells. These findings highlight the potential antitumor activity of EGCG, particularly in reducing the viability of cells within a 3D tumor-like microenvironment. These results reinforce the requirement for cell culture models that best resemble the *in vivo* environment. Besides, the effect of EGCG on genes responsible for cell adhesion justifies further studies on its impact on tumor cells, perhaps with simultaneous treatment with chemotherapeutic drugs.

## Supplementary Material





## Notes

Supplementary Table 1 listing gene expression matrix used for Figure 2 heatmap; classification of cells by cell source type, cell lineage/type, tissue, and disease distribution by cluster (XLSX)
Supplementary Figure 1 showing distribution of cell types across clusters (PDF)

## §

Genetics Division, Department of Morphology and Genetics, Universidade Federal de São Paulo, Botucatu St. 740, Vila Clementino, 04023-062 São Paulo, SP, Brazil
