# Supplementary material for: Unraveling the Effects of Epigallocatechin-3-gallate on Hepatocellular Carcinoma Cells: A Comparative Analysis of Monolayer vs Multicellular Tumor Spheroids
Source: ACS Omega. 2025 Jul 31;10(31):34238–53. doi: 10.1021/acsomega.5c00839 (PMC12355261; doi:10.1021/acsomega.5c00839)
Supplement: Supplementary file 2 [file ao5c00839_si_002.pdf]

Unraveling the effects of epigallocatechin-3-gallate on hepatocellular carcinoma cells: a comparative analysis of monolayer vs. multicellular tumor spheroids

Mariana dos Reis Simpronio[†]

Ana Rita Thomazela Machado[†]

Patrick Santos[†]

Diego Luis Ribeiro[‡]

Lusânia Maria Greggi Antunes[†]

Alexandre Ferro Aissa[†*]

[†]Department of Clinical Analysis, Toxicology and Food Sciences, School of Pharmaceutical Sciences of Ribeirão Preto, University of São Paulo - USP, Ribeirão Preto, SP, Brazil.

‡Department of Genetics, Ribeirão Preto Medical School, University of São Paulo - USP, Ribeirão Preto, SP, Brazil

[*]Corresponding author: Alexandre Ferro Aissa

Present Address for AFA: e-mail: aissa@unifesp.br

Genetics Division, Department of Morphology and Genetics, Universidade Federal de São Paulo, Botucatu St. 740, Vila Clementino, 04023-062, São Paulo, SP, Brazil.

Supplementary Table 1. Table of gene expression used to create the heatmap in Figure 1, as well as the classification of cells according to Cell Source Type, Cell Lineage/Type, Tissue, and Disease Distribution by cluster.

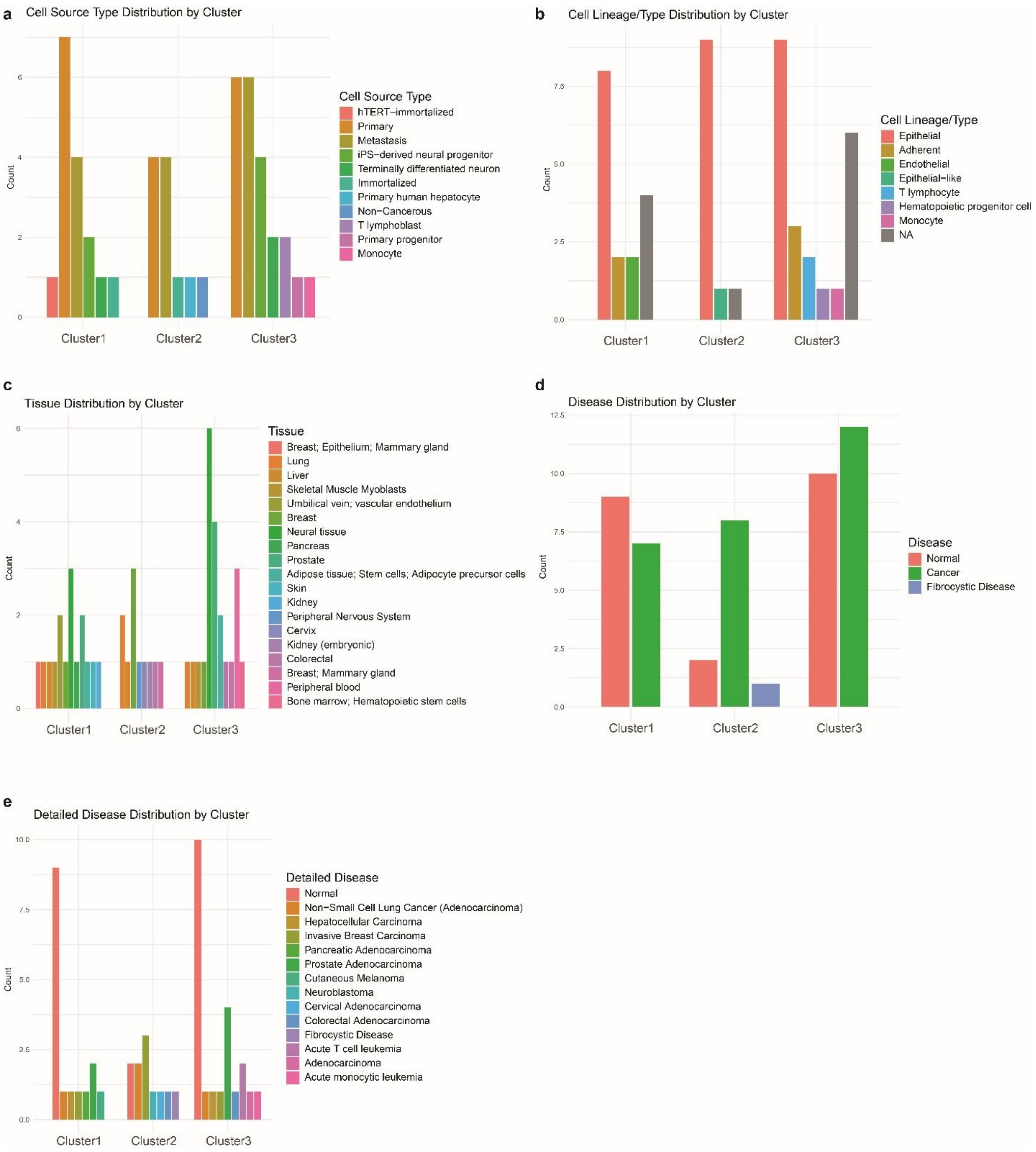

Supplementary Figure 1. Distribution of cell types across clusters. a) Cell Source Type Distribution by Cluster: Bar plot showing the count of cells in each cluster categorized by their source type, including primary cells, immortalized lines, and metastasis-derived cells.
b) Cell Lineage/Type Distribution by Cluster: Distribution of cell lineage or type (e.g., epithelial, endothelial, T lymphocyte) within each cluster, highlighting cellular origins.
c) Tissue Distribution by Cluster: Representation of tissues of origin for cells in each cluster.
d) Disease Distribution by Cluster: Categorization of cells by associated diseases and normal conditions.
e) Detailed Disease Distribution by Cluster: Expanded view of disease categorization, providing finer details for conditions present in the dataset.
